# Effect of Receiving Financial Support from Adult Children on Depression among Older Persons and the Mediating Role of Social Participation

**DOI:** 10.3390/ijerph191912974

**Published:** 2022-10-10

**Authors:** Wenran Xia, Jeroen D. H. van Wijngaarden, Robbert Huijsman, Martina Buljac-Samardžić

**Affiliations:** Erasmus School of Health Policy and Management, Erasmus University Rotterdam, 3062 PA Rotterdam, The Netherlands

**Keywords:** financial support, social participation, depression, mediator, older persons

## Abstract

Older persons are vulnerable to depression SFduring the ageing process. Financial resources and social participation are expected to have an impact on depressive symptoms. This study investigated the relationship between financial support from children and depression among Chinese older persons, as well as the mediating effect of social participation in this relationship. Data from 7163 participants aged 60 and above were extracted from wave 2015 and 2018 of the China Health and Retirement Longitudinal Survey (CHARLS). A multivariate regression analysis was performed on both cross-sectional data and two-wave longitudinal data to test our hypotheses. The results revealed that financial support from children was negatively associated with depressive symptoms in both the short-term and the long-term. In addition, this relationship was partially mediated by social participation in the short-term association and fully mediated by social participation in the long-term, where financial support was positively related to social participation, and social participation was negatively associated with depressive symptoms. This study offers an in-depth insight into the relationship between financial support from children and depression among Chinese older persons. Policies and initiatives to stimulate social participation should be promoted to improve older persons’ mental health.

## 1. Introduction

Population ageing is one of the most important medical and socioeconomic challenges worldwide. According to China’s seventh census in 2020, the population of people aged 60 and above was 264.2 million, which accounts for 18.70% of the total population [1]. Depression is a common mental disorder for older persons that is becoming an important issue along with accelerated ageing. According to a recent study based on an investigation of 22 locations in China, over 17% of males and 23% of females aged 60 and above were found to have depressive symptoms [2]. It has been reported that the disease burden caused by depression is increasing: the burden in 2016 was 1.7 times higher than that in 2000 [3]. Depression harms the quality of life, reduces life satisfaction, and even increases the incidence of suicide [4]. Research suggests that financial resources and social participation are important factors explaining depression [5,6]. We expect that the financial support of adult children, as an expression of filial piety, is important in alleviating financial stress and helps to stimulate social participation, thereby having a positive effect on the mental health of Chinese older persons.

### 1.1. Financial Support and Depression

Studies have shown that financial stress is a strong predictor of depression among older adults [5,7]. For example, recent studies conducted during the COVID-19 period found that financial strain is significantly associated with more depressive symptoms [8,9]. Although the economy has grown rapidly in China, not everybody benefits equally, and inequality is increasing [10]. Poverty is still prevalent among Chinese older persons, and even those who receive a pension can often only meet their basic needs. As a consequence, many older persons, especially those in rural areas, have to continue working to sustain themselves [11]. The low incomes expose them to high levels of stress and lead to poor psychological health, especially in the face of possible life crises [12].

Social support was found to be a positive factor for depression in later life [13]. Economic support such as pensions was found to improve depression through a change of lifestyle, increasing health investments, and economic security due to reduced financial hardship [14]. If individuals receive practical and emotional support from their social network, they will be more effective in utilizing their resources and be less prone to suffer from physical and psychological issues [7]. In the network of social support, support from family was rated to be more important than other types of support, such as support from community and government [15]. Financially speaking, receiving support from adult children may alleviate financial stress, improve nutrition and enable older persons to cope better financially with setbacks [16]. 

There are different opinions on the impact of financial support from children on older persons [17,18,19]. Some believe that receiving financial support creates a sense of guilt and feelings of being useless because older persons may feel that they are a burden to their children. Studies in several European Mediterranean countries, for example, found no positive associations between receiving money and mental health for older persons. It was, therefore, suggested that receiving money from children is accompanied by a sense of shame in Mediterranean culture [17]. Some others, however, believe that financial support from adult children improves older persons’ quality of life and helps them obtain a sense of safety, which reduces the risk for depression. It is common for adult children to provide financial assistance to their older parents in more traditional societies and cultures. Arab older persons are more likely to receive financial support from children, and this is associated with greater positive effects, such as well-being, than in the case of Jewish older persons. This shows that culture is an important force in predicting what type of intergenerational support is expected and accepted [18]. Chinese children are expected to support their older parents and ensure their basic living needs as an expression of filial piety. The traditional value of filial piety in China contains a series of expected duties of children, including respect, obedience, loyalty, material provision and physical care to their parents [20]. Studies show that providing material support, including financial support, to older parents is one way to demonstrate filial piety [19], and older persons who are satisfied with their children’s filial piety reported a higher level of psychological comfort [21]. Although studies have shown that family support has a significant impact on mental health, few studies have explored the specific role of financial support from children [22,23]. The first aim of this study is, therefore, to investigate the relationship between financial support that older persons receive from adult children and mental health.

**Hypothesis** **1.** *The financial support that older persons receive from adult children will be positively associated with their mental health, as indicated by a negative relationship between the lack of financial support with a high occurrence of depressive symptoms.*

### 1.2. Social Participation and Depression

Social participation is broadly defined as ‘the conscious and active engagement in outdoor social activities leading to interacting and sharing resources with others, and personal satisfaction resulting from that engagement’ [24]. It is believed to play a key role in improving mental health [25,26] for urban and especially rural older persons [27]. According to activity theory, older persons have the same psychological and social needs as middle-aged individuals. Consequently, as they withdraw from society, older individuals may experience a loss of well-being, low self-esteem and isolation. Therefore, older persons are more likely to achieve successful ageing if they continue to be active after retirement [28]. Productive roles are expressed through participation in different social activities. For example, playing mahjong or cards and enjoying sports, social clubs and interacting with friends are associated with fewer depressive symptoms for Chinese urban older persons [29]. Evidence from European countries has shown that social participation behavior such as participation in religious organizations, volunteering and altruistic behavior are beneficial for older persons’ mental health [30,31,32,33]. Based on these findings, we pose the following hypothesis:

**Hypothesis** **2.** *Participating in social activities will be positively associated with older persons’ mental health; specifically, a higher level of social participation will be associated with fewer depressive symptoms.*

### 1.3. Financial Support, Social Participation and Depression

Social participation is influenced by the resources that one possesses. According to continuity theory, individuals experience withdrawal during the process of ageing. As an adaptive strategy, older adults attempt to make use of available resources to preserve and maintain a sense of continuity and stability and maintain their social roles. As individuals’ resources and abilities increase, their ability to continue in social roles increases [34]. Older persons with high socioeconomic status and corresponding resources can maintain previous social roles much more easily than those lacking socioeconomic status and resources [35]. Mood [36] found that poverty negatively influenced social participation among Swedish adults. Similarly, Feng [37] found that older persons from a higher-income group were almost two times more likely to participate in social activities than those from a lower-income group. As an expression of filial piety, financial support from adult children is an important economic resource for Chinese older persons, especially for those with a lower economic status. Although research has been conducted on the relationship between socioeconomic position and social participation, there is still no study on how receiving financial support from adult children affects older persons’ social participation behavior. We expect that older persons who receive financial support from adult children experience less financial stress, which allows them to participate more in social activities, as more leisure time and means are available. The third hypothesis is, therefore:

**Hypothesis** **3.** *Financial support from adult children will be positively associated with the intensity of social participation of the older persons.*

It has been claimed that financial stress may affect depression through social pathways [5]. Studies found, for example, that the burden of depressive symptoms due to financial strain in earlier life may be attenuated by social engagement in later life [38]. However, whether social participation also impacts the relationship between depressive symptoms and financial status in later life is unknown. We expect that financial support from children may not only alleviate the financial stress of Chinese older persons whose pension level is still relatively low, but also allow older persons to spend more time on social activity, which satisfies their need to connect to society, reduces the risk of social isolation, and finally, prevents depression. Based on the foregoing arguments, we pose our final hypothesis:

**Hypothesis** **4.** *Social participation mediates the relationship between received financial support and mental health among Chinese older persons. Specifically, receiving more financial support from children is related to higher levels of social participation, and a higher level of social participation is further related to lower levels of depression.*

## 2. Materials and Methods

### 2.1. Data Source and Sample

The data in the present study are derived from the China Health and Retirement Longitudinal Survey (CHARLS), which is conducted by the National School of Development (China Centre for Economic Research) of Beijing University and contains a nationally representative sample of Chinese adults aged 45 years old and above and their spouses. The response rate was high at 87.15% in wave 2015 and 86.46% in wave 2018 [39,40]. The PPSS (probability-proportional-to-size sampling) and CAPI technology (computer-assisted personal interviewing) were adopted to randomly collect multistage samples (county/district-village/community-household) [39]. The baseline wave survey was conducted in 2011 and includes over 17,500 individuals in 150 counties/districts and 450 villages/resident committees from 28 provinces in China. The follow-up waves of the survey are conducted every two years, and data are made public one year after the end of data collection.

**Figure 1:**
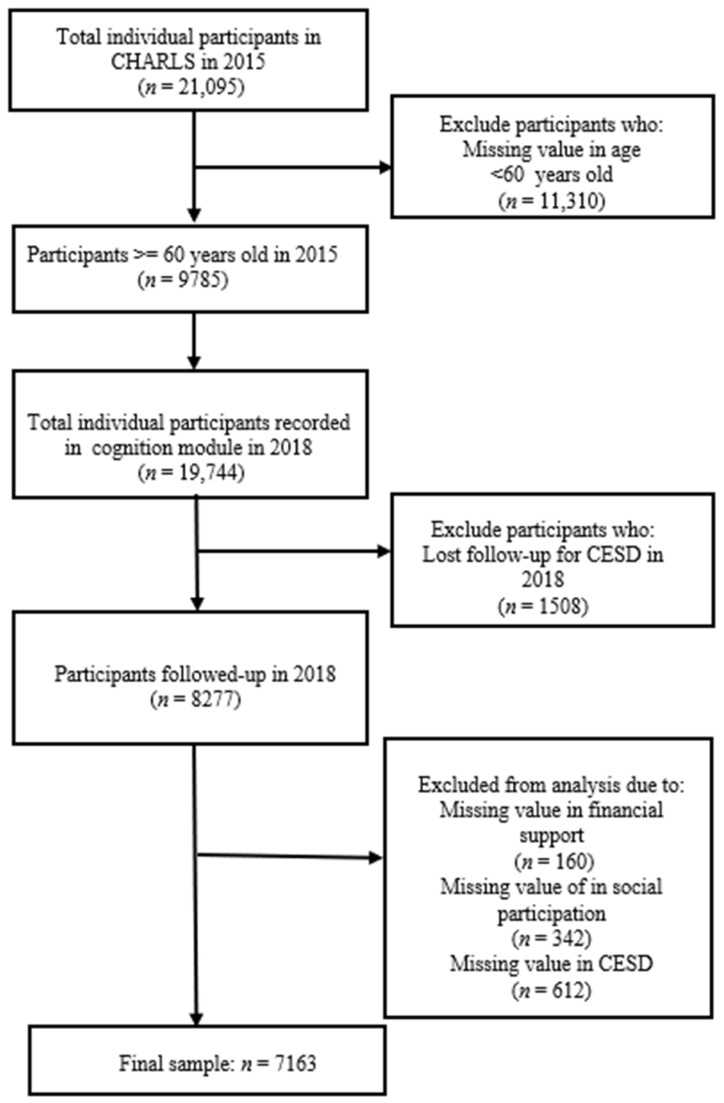
Flow chart of sample selection.

Data from 2015 and 2018 were used in the present study. We restricted our attention to respondents aged 60 and above and those who had records both in wave 2015 and wave 2018. Respondents who had missing values in the key variables of financial support, social participation and depressive symptoms were excluded. Finally, 7163 older persons were selected for the analysis (Figure 1). 

The CHARLS obtained approval for interviewing the respondents and collecting data from the Biomedical Ethics Review Committee of Peking University (IRB00001052-11015), and the respondents were asked to sign informed consent forms. Hence, additional ethics approval was not needed.

### 2.2. Measurements

#### 2.2.1. Depressive Symptoms

Depressive symptoms in both 2015 and 2018 were measured using the 10-item Center for Epidemiological Studies Depression (CES-D) scale [41]. This scale has been shown to have good reliability and validity among community-residing older adults (Cronbach’s alpha = 0.81) [42] as well as in this study (Cronbach’s alpha = 0.80). Individuals were asked about the frequency of depression-related feelings and behaviors during the last week with four available options on 10 depressive symptom-related questions (0 = Rarely or none of the time, 1 = Some or a little of the time, 2 = Occasionally or a moderate amount of the time, 3 = Most or all of the time). Answers to all the items were summed, producing a score ranging from 0 to 30. The higher the scores are, the more depressive symptoms a participant has.

#### 2.2.2. Social Participation

The indicator of social participation was obtained through two questions. First, “Have you done any of these activities in the last month?” with 12 available options: (1) interacted with friends; (2) played mahjong, played chess, played cards, or went to the community club; (3) provided help to family, friends, or neighbors who did not live with you; (4) went to a sport, social, or other kinds of club; (5) took part in a community-related organization; (6) engaged in voluntary or charity work; (7) cared for a sick or disabled adult who did not live with you; (8) attended an educational or training course; (9) stock investment; (10) used the internet; (11) other; and (12) none of these. Participants who answered ‘yes’ for the first 11 questions were then asked about the attending frequency for each social activity in the last month. The answers were as follows: (1) almost daily; (2) almost every week; and (3) not regularly. We reverse-coded the answers and included participants who had never attended any type of activity. Hence, the code of frequency for each social activity was 0 (never); 1 (not regularly); 2 (almost every week); and 3 (almost daily); and the frequency of each activity was summed to produce the score of social participation intensity; a higher score indicates a higher level of social participation.

#### 2.2.3. Financial Support

The variable financial support includes two categories, economic transfers and in-kind transfers from non-coresident children in the past year. Economic transfers include living expenses such as water, electricity, telephone rates and loans, and other regular and irregular costs. In-kind transfers include food, clothes, and other regular and irregular material support. All transfers were calculated in Chinese Renminbi Yuan (CNY 7 = USD 1). The sum value of all children’s support for financial and in-kind transfers was calculated, and the logarithm of the sum was taken, the same as in related studies [43,44].

#### 2.2.4. Covariates

Three categories of covariates were controlled. First, we included demographic factors that have been shown to have impacts on depression and social participation [45,46], including age (continuous variable), gender (1 = male, 0 = female) and marital status (1 = married, 0 = others). Education level was coded as lower (lower than elementary school) and higher (elementary school and above). The registration information of Chinese citizens could be determined according to their living place and hukou. Hukou refers to the household registration system that assigns each citizen an agricultural or non-agricultural status. This affects the resources available to them including housing, employment, education and healthcare services [47]. For example, residents with an urban hukou have a higher reimbursement of inpatient visit and medical treatment [48,49]. Taking this into account, we controlled for both living area (1 = rural, 0 = urban) and hukou status (agricultural hukou/non-agricultural hukou) as the type of residence. Since the questionnaire only asked about the financial support from non-coresident children, we also controlled for whether respondents live with children (yes = 1, no = 0). Socioeconomic factors were also included. Work status is a dichotomous variable that indicates whether participants are currently working (1 = yes, 0 = no). Although many studies used household consumption as a socioeconomic factor, it may be partially overlapped with our independent variable. Given that studies indicate pension as a significant financial factor that influences older adults’ mental health [50,51], the logarithm of yearly total pension income (continuous variable) was controlled to specifically test the effect of intergenerational financial support. Previous studies have shown that a significant factor of mental health is health status [52]. In our study, the ability of daily living (ADL) limitations was coded as 1 if respondents had limitations for at least one of the following activities: dressing, bathing, eating, getting into or out of bed, using the toilet, and controlling urination and defecation. Chronic disease was coded as ‘yes’ if respondents reported having any of the following diseases: hypertension, dyslipidemia, diabetes or high blood sugar, cancer or malignant tumor, chronic lung diseases, liver disease, heart disease, stroke, kidney disease, stomach or other digestive diseases, emotional/nervous/psychiatric problems, memory-related disease, arthritis and asthma. Finally, self-rated health (SRH) was evaluated by asking respondents how they would rate their health status on a 5-point Likert scale: 1 = very good, 2 = good, 3 = fair, 4 = poor and 5 = very poor. In accordance with previous studies, we set the cut-off point at 3 and made it a dichotomous variable; scores of 1 or 2 were classified as ‘good’, while others were grouped as ‘poor’ (good = 1, poor = 0) [53]. Finally, to exclude the effects of the dependent variable itself across time, we also included depressive symptoms in 2015 as covariates in the long-term analysis.

### 2.3. Statistical Analysis

**Figure 2:**
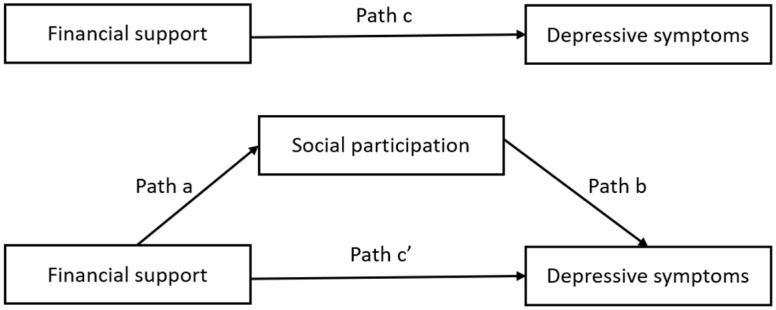
Conceptual framework of the present study.

A descriptive statistical analysis was conducted before running the regression. The multicollinearity test among independent variables shows that the variance inflation factor (VIF) was between 1.02 and 1.89, and the tolerance value was greater than 0.76. This is well within the acceptable range, indicating that no multicollinearity exists between the independent variables. Bivariate correlation analyses were conducted to test the associations between variables. To test our hypotheses, we performed a cross-sectional analysis based on data from 2015 and a two-wave longitudinal analysis using data from 2015 and 2018. In the longitudinal analysis, the independent variable and mediator were extracted from wave 2015, considering that receiving resources is more likely to impact social participation in a relatively short period of time [54]. Depression symptoms in 2018 served as a dependent variable, and those in 2015 were included as one of the covariates. This allowed us to test the short-term and long-term effect of financial support on depressive symptoms as well as the mediating effect of social participation. Meanwhile, the cross-wave analysis provides stronger evidence of a causal relationship. As shown in Figure 2, except for exploring the total effect of financial support on depressive symptoms (path c), we tested unstandardized regression coefficients for path a (path from financial support to social participation); path b (from social participation to depressive symptoms); the direct effect of financial support on depressive symptoms (path c); and their indirect effect after controlling for social participation using bootstrapping procedures.

Multivariate regression and mediation analyses were conducted with the support of PROCESS 4.0 [55]. The bootstrapping procedure was shown to overcome the limitations of Baron and Kenny’s causal steps approach and Sobel’s test and was less affected by the sample size and the distribution of samples. It was also able to produce more accurate results [56]. In this study, each of the 5000 bootstrap samples was used to test the mediating effect, as in other studies [16,57]. Bias-corrected 95% confidence intervals (Cis) were used to investigate the mediation effect, and the mediating effect was considered significant if 0 was not located in the CI range. All of the covariates identified above were included in these analyses. We used multiple imputations by chained equations (MICE) to impute missing values for covariates. All analyses in this study used R software package, version 4.1.2 [58]. Multiple imputations for missing data were performed by using the ‘mice’ package, and a mediation analysis was performed by PROCESS in R, which was developed by Hayes. *p* < 0.05 was considered statistically significant.

## 3. Results

### 3.1. Sample Description

The demographic characteristics of all participants are presented in Table 1. The average age of the participants was 67.36 years old (SD = 6.00); 48,8% of the respondents were male, and most (82%) of the respondents were married. Over half (52.5%) of the respondents had a lower education level. Although there was a slight difference, the respondents lived in rural areas or had an agricultural hukou account for about 75% of the total sample, which indicates the reliability of both variables. A total of 72.5% of the respondents had at least one kind of chronic disease, while over 70% of them did not have any ADL limitations, and nearly 90% of them reported good health status. The average amount of financial support from children was 3.01 (SD = 1.31), more than the amount of pension that the respondents received, which was 2.31 (SD = 1.60). The strength of social participation was 1.65 (SD = 2.22). The mean value of the CES-D score was 8.24 (SD = 6.50) in 2015 and 8.75 (SD = 6.60) in 2018.

### 3.2. Bivariate Relationship among Key Variables

A bivariate correlation analysis was performed to test the relationship between the variables. As presented in Table 2, the results showed that financial support was positively associated with social participation (r = 0.05, *p* < 0.001) but not significantly associated with depressive symptoms in 2015 (r = −0.02, *p* > 0.05) and 2018 (r = −0.02, *p* > 0.05). Social participation is negatively associated with depressive symptoms in both 2015 (r = −0.13, *p* < 0.001) and 2018 (r = −0.12, *p* < 0.001). It is notable that both living area and hukou had a relatively strong correlation with key variables with similar coefficients. Specifically, respondents who lived in urban areas or had a non-agricultural hukou received a higher level of financial support (r = 0.08, *p* < 0.001 for both variables). Similarly, social participation intensity was higher for those who lived in urban areas (r = −0.21, *p* <0.001) or had non-agricultural hukou. Finally, living in a rural area or having an agricultural hukou was positively related to depressive symptoms in 2015 (r = 0.14, *p* < 0.001 for both variables); similar patterns were also found in 2018 (r = 0.12, *p* < 0.001 for living area, r = 0.14, *p* < 0.001 for hukou). These results indicate significant relationships between variables, although the strength of the correlation is weak.

### 3.3. Cross-Sectional Mediating Effect

We first tested the relationship between financial support, social participation and depressive symptoms with one-wave data. In this analysis, all variables were extracted from wave 2015. The results are indicated in Table 3. After controlling for all covariates, the result in path c showed that financial support was negatively associated with depressive symptoms (β = −0.20, *p* < 0.001). Financial support had a positive association with the intensity of social participation (β = 0.12, *p* < 0.001), indicating a beneficial role of receiving financial support from children in social participation. Meanwhile, social participation showed a negative association with depressive symptoms (β = −0.20, *p* < 0.001), while controlling for financial support. The mediation analysis showed a significant indirect effect of social participation, revealing a mediating role of social participation on the relationship between financial support and depressive symptoms (β = −0.02, 95% CI [−0.04, −0.02]). In addition, the direct effect of financial support on depressive symptoms weakened but stayed significant after accounting for the mediator (β = −0.18, 95% CI [−0.28, 0.07]). Generally speaking, these results showed a partial mediating effect of social participation in the short-term relationship between received financial support and depressive symptoms That is, financial support positively affects depressive symptoms directly and via social participation, meaning that it positively influences social participation, and social participation, in turn, negatively affects depressive symptoms.

### 3.4. Longitudinal Mediating Effect

We also tested the mediating relationship with longitudinal data. The results are shown in Table 4. In path c, financial support in 2015 negatively affected depressive symptoms in 2018, although the effect size has decreased (β = −0.10, *p* < 0.05). Similar to the results in the previous analysis, financial support positively affected social participation (β = 0.19, *p* < 0.001). Social participation in 2015 had a negative association with depressive symptoms in 2018 (β = −0.09, *p* < 0.01), while controlling financial support and depressive symptoms in the past (2015). The mediation analysis showed a significant indirect effect of social participation and revealed a mediating role of social participation on the relationship between financial support and depressive symptoms (β = −0.01, 95% CI [−0.02, −0.004]). It is worth noting that the direct effect of financial support on two-year-later depressive symptoms became insignificant after accounting for social participation (β = −0.09, 95% CI [−0.19, 0.01]). These results indicate a full mediating effect of social participation in the long-term negative relationship between financial support and depressive symptoms. That is, financial support positively affects social participation, and social participation, in turn, negatively affects depressive symptoms.

## 4. Discussion

This study contributes several important findings toward understanding the relationship between intergenerational financial support and depression among Chinese older persons. First, our results suggest that receiving financial support and social participation leads to fewer depressive symptoms in both the short and long term. Importantly, we found a partial mediating effect of social participation in the short-term relationship between financial support and depressive symptoms, and this partial mediation turned into full mediation in the long-term relationship between financial support and depressive symptoms. In other words, receiving financial support from adult children is beneficial for social participation, and social participation, in turn, alleviates depressive symptoms.

We found a significant negative impact of financial support from children on both short-term and long-term depressive symptoms among Chinese older persons, which confirmed our first hypothesis. This is in line with the finding of [43] that receiving financial support from children is mentally beneficial for older parents, although this study took household expenditure as the proxy of economic status that may incorporate different types of income, including pension, which is the largest source of income inequality among older-person households in China [59]. Our finding indicates that financial resources, particularly from adult children, may alleviate the financial stress of older persons and, thus, decrease their depressive symptoms. This suggests that the beneficial effect of intergenerational financial support is relevant in the short and long run. It is notable that in recent years, children’s obligation to their parents has been redefined as the processes of fast industrialization and urbanization have occurred. Although there is a positive influence of intergenerational financial support, there is also a downside. Some studies in Chinese communities such as Hong Kong and Singapore found that financial support sometimes creates feelings of being a burden and results in excessive guilt and shame among older persons, similar to findings in other developed countries [60,61]. In addition, the development of new communication technology enables adult children to provide emotional support to their older parents without living nearby. Future studies are needed to update the meaning of financial support to Chinese older persons in terms of both financial and cultural dimensions.

Consistent with our second hypothesis, we found a negative impact of social participation on both short-term and long-term depressive symptoms. This result is consistent with previous findings that social participation leads to better mental health and participating in social activities consistently promotes individuals’ well-being in later life [62]. This finding supports the activity theory of ageing that through interacting with friends as well as other social activities, older persons build social engagement and share resources with others, which helps them to receive more socio-emotional support, and thus, improves their mental health [63].

Finally, we found a mediating role of social participation in the relationship between financial support and depressive symptom, confirming our Hypothesis 3 and 4. This seems to be in line with other findings that social participation mediates the relationship between socioeconomic status and mental health [52]. Sufficient financial resources allow individuals to spend more time and energy participating in various social activities. Activities such as playing mahjong, attending an educational course, online shopping, and even simply interacting with friends are all leisure activities that require financial or time investment. Other studies also found that income and pension positively influence the social engagement of older persons [37,64]. However, Feng [37] did not differentiate between different sources of income, and Zhu [64] only focused on pension income. Our study showed that even after controlling for pension income, the financial support from adult children still has a positive effect on social participation, emphasizing the beneficial effect of intergeneration financial resources. The results of the cross-sectional analysis showed a partial mediating role of social participation as well as a significant direct effect of financial support and depression. This suggests that financial support benefits mental health not only by decreasing individuals’ financial stress, but also by stimulating social participation. Interestingly, this partial mediating effect turned into a full mediation in the long-term impact of financial support on depressive symptoms. A likely explanation for this is that financial support may reduce the financial stress of older persons and thereby improve their psychological well-being for a certain period. However, this direct benefit may deteriorate over time after the money is spent. In contrast, as an adaptive strategy for older persons to maintain their previous social roles, participating in social activities helps individuals to connect to their social networks and may prevent them from social isolation over a longer period of time [65]. Hence, it is no longer the physical benefits or sense of financial safety that influences depressive symptoms in older persons, but rather the behavior of social participation that financial support facilitates, which is consistent with the continuity theory [34]. Generally, both the results in the short term and long term suggest that social participation is important for Chinese older persons for alleviating depression. 

It should be noted that the present study took the intensity of all types of social activities that older persons attended as the indicator of social participation without testing the separate effects of each form of social participation. Research has found that older persons in a Western cultural context are more likely to engage in social activities independently, such as volunteering, than take part in activities with high involvement with others. Eastern cultures may prefer to interact with friends and depend on each other, given that the core unit of survival is the group [66,67]. In recent times, Internet use has become a popular form of social participation for older persons, and activities such as chatting with friends or shopping online are widely replacing traditional social activities [68,69]. Therefore, different types of social participation may have different impacts on the health outcome of older persons across cultures. Further studies could be performed to explore the specific role of different forms of social participation on the relationship between intergenerational financial support and mental health.

### 4.1. Limitations

Several limitations should be noted in this study. First, although financial support from family members and pensions are important financial sources for Chinese older persons, not all income sources, such as other earnings, were included. Future studies could be performed to test the specific role of different financial resources on mental health among older persons. Additionally, living area and hukou are outstanding covariates in our result, indicating that the resident place and hukou status are significant factors of financial support, social participation and depressive symptoms of older persons. Many previous studies found rural–urban disparity in health-related behavior and health outcomes among older persons. For example, the prevalence of depressive symptoms is higher among older persons residing in rural areas than those living in urban areas, and the health effect of social participation is stronger for rural older persons too [27,70]. Older persons living in rural areas have more unmet needs than those residing in urban areas, even though they comprise the majority of the aged population in China [71]. Future studies could be conducted to further investigate the role of rural–urban differences in the relationship between financial support, social participation and depression among older persons, but also particularly the needs of rural older persons. Finally, although the dataset we used in this study had a high response rate [40], the response bias still exists, since not all targeted people participated in the survey.

### 4.2. Implications

Despite these limitations, our study provides important signals for health-related policymakers and professionals to provide better care for older persons. We found a beneficial role of financial support on depressive symptoms among Chinese older persons both in the short and long run. It is important for policymakers to strive to improve the economic well-being of older persons and ensure they have stable financial resources. Moreover, our study emphasized the important beneficial role of social participation in the relationship between financial support from children and depressive symptoms, and practitioners working in the community and in mental health services are encouraged to construct interventions to stimulate social participation among older persons, and thus, benefit their mental health.

## 5. Conclusions

The present study revealed that receiving financial support from adult children has a negative association with depression among Chinese older persons, although this effect diminishes over time. More importantly, social participation serves as a mediator through which financial support benefits the alleviation of depressive symptoms in both the short and long run. We suggest that, in addition to ensuring financial resources, it is even more important that policies and initiatives be proposed by policymakers and related professionals to promote social participation among older persons.

## Figures and Tables

**Table 1:** Sociodemographic characteristics (N = 7163).

Variables	N (%)	Mean (SD)	Range
Age		67.36 (6.00)	60–93
Gender			
Male	3501 (48.88)		
Female	3662 (51.12)		
Marital status			
Married	5914 (82.56)		
Not married	1249 (17.44)		
Living area			
Rural	5258 (73.40)		
Urban	1905 (26.60)		
Hukou			
Agricultural hukou	5576 (77.84)		
Non-agricultural hukou	1587 (22.16)		
Whether coresident with children			
Yes	3529 (49.27)		
No	3634 (50.73)		
Education			
Lower	3762 (52.52)		
Higher	3401 (47.48)		
Work status			
Yes	4058 (56.65)		
no	3105 (43.35)		
Pension income (log scale)		2.31 (1.60)	0–5.46
Ability of daily living limitation			
Yes	1729 (24.24)		
No	5434 (75.86)		
SRH			
good	6374 (88.99)		
poor	789 (11.01)		
Whether have chronic disease			
Yes	5185 (72.39)		
No	1978 (27.61)		
Depressive symptoms (2015)		8.24 (6.50)	0–30
Financial support (log scale)		3.01 (1.31)	0–5.88
Social participation		1.65 (2.22)	0–15
Depressive symptoms (2018)		8.75 (6.60)	0–30

**Table 2:** Correlation among variables.

	1	2	3	4	5	6	7	8	9	10	11	12	13	14	15	16
1. Age	1															
2. Gender	0.03 *	1														
3. Marital status	−0.26 ***	0.18 ***	1													
4. Living area	−0.02	0	0	1												
5. Hukou	−0.07 ***	−0.07 ***	−0.02 *	0.64 ***	1											
6. Co-residence	−0.06 ***	−0.01	−0.08 ***	0.02 *	0.08 ***	1										
7. Education	−0.06 ***	0.32 ***	0.11 ***	−0.27 ***	−0.33 ***	−0.05 ***	1									
8. Work	−0.27 ***	0.13 ***	0.14 ***	0.34 ***	0.34 ***	0.02	−0.09 ***	1								
9. Pension	0.13 ***	0.05 ***	0	−0.15 ***	−0.19 ***	−0.05 ***	0.11 ***	−0.09 ***	1							
10. ADL limitation	0.12 ***	−0.1 ***	−0.07 ***	0.07 ***	0.07 ***	0	−0.12 ***	−0.1 ***	−0.02	1						
11. SRH	0.03 **	−0.04 ***	−0.02	0.03 *	0.02	0.03 *	0	−0.03 **	0.01	0.13 ***	1					
12. Chronic disease	0.04 **	−0.06 ***	−0.01	0.04 ***	0.04 ***	−0.02	−0.05 ***	−0.04 ***	−0.01	0.13 ***	0.13 ***	1				
13. Depressive symptoms (2015)	0.01	−0.19 ***	−0.11 ***	0.14 ***	0.14 ***	0.04 **	−0.18 ***	0.01	−0.05 ***	0.33 ***	0.19 ***	0.15 ***	1			
14. Financial support	0.04 **	−0.03 *	0	0.08 ***	0.08 ***	−0.02	−0.04 **	0.02	0.02	0.03 *	−0.01	0.05 ***	−0.02	1		
15. Social participation	−0.02 *	0.02	−0.02	−0.21 ***	−0.23 ***	−0.03 **	0.19 ***	−0.13 ***	0.12 ***	−0.06 ***	−0.06 ***	−0.02	−0.13 ***	0.05 ***	1	
16. Depressive symptoms (2018)	−0.01	−0.18 ***	−0.07 ***	0.12 ***	0.14 ***	0.03 **	−0.18 ***	0.03 **	−0.08 ***	0.25 ***	0.16 ***	0.14 ***	0.52 ***	−0.02	−0.12 ***	1

* *p* < 0.05, ** *p* < 0.01, *** *p* < 0.001.

**Table 3:** Cross-sectional mediating effect of social participation in the association between financial support and depressive symptoms.

Variables	Path c				Path a				Path b/c’			
	B	SE	LLCI	ULCI	B	SE	LLCI	ULCI	B	SE	LLCI	ULCI
Age	−0.04 **	0.01	−0.06	−0.01	−0.02 ***	0.00	−0.03	−0.01	−0.04 ***	0.01	−0.07	−0.02
Gender	−1.40 ***	0.15	−1.70	−1.10	−0.09	0.05	−0.20	0.02	−1.42 ***	0.15	−1.71	−1.12
Marital status	−1.16 ***	0.19	−1.55	−0.78	−0.28 ***	0.07	−0.42	−0.15	−1.22 ***	0.19	−1.60	−0.84
Living area	0.76 ***	0.21	0.35	1.17	−0.35 ***	0.08	−0.50	−0.20	0.69 **	0.21	0.28	1.10
Hukou	0.78 ***	0.23	0.34	1.23	−0.65 ***	0.08	−0.81	−0.48	0.65 **	0.23	0.21	1.10
Co-residence	0.17	0.14	−0.11	0.44	−0.07	0.05	−0.17	0.03	0.16	0.14	−0.12	0.43
Education	−0.96 ***	0.16	−1.27	−0.65	0.55 ***	0.06	0.44	0.66	−0.85 ***	0.16	−1.16	−0.54
Work	0.15	0.16	−0.16	0.46	−0.24 **	0.06	−0.36	−0.13	0.10	0.16	−0.21	0.41
Pension	−0.05	0.04	−0.14	0.04	0.10 ***	0.02	0.07	0.13	−0.03	0.04	−0.12	0.06
ADL limitation	4.23 ***	0.17	3.90	4.56	−0.14 *	0.06	−0.26	−0.02	4.21 ***	0.17	3.88	4.53
SRH	2.75 ***	0.23	2.31	3.19	−0.36 ***	0.08	−0.52	−0.20	2.68 ***	0.23	2.24	3.12
Chronic disease	1.24 ***	0.16	0.93	1.55	0.02	0.06	−0.09	0.13	1.24 ***	0.16	0.93	1.55
Financial support	−0.20 ***	0.05	−0.31	−0.10	0.12 ***	0.02	0.09	0.16	−0.18 ***	0.05	−0.28	−0.07
Social participation									−0.20 ***	0.03	−0.27	−0.14
Constant	7.97 ***	0.97	6.07	9.86	3.85 ***	0.35	3.16	4.53	8.74 ***	0.97	6.83	10.65
R^2^	0.19				0.09				0.19			

* *p* < 0.05, ** *p* < 0.01, *** *p* < 0.001.

**Table 4:** Longitudinal mediating effect of social participation in the association between financial support and depressive symptoms.

Variables	Path c				Path a				Path b/c’			
	B	SE	LLCI	ULCI	B	SE	LLCI	ULCI	B	SE	LLCI	ULCI
Age	−0.02	0.01	−0.04	0.01	−0.02 ***	0.00	−0.03	−0.01	−0.02	0.01	−0.04	0.00
Gender	−0.89 ***	0.14	−1.17	−0.60	−0.13 *	0.06	−0.24	−0.02	−0.90 ***	0.14	−1.18	−0.62
Marital status	−0.14	0.18	−0.50	0.22	−0.31 ***	0.07	−0.45	−0.18	−0.17	0.18	−0.53	0.19
Living area	0.22	0.20	−0.17	0.61	−0.31 ***	0.08	−0.46	−0.16	0.19	0.20	−0.20	0.58
Hukou	0.27	0.21	−0.15	0.69	−0.65 ***	0.08	−0.81	−0.49	0.21	0.22	−0.21	0.63
Co-residence	0.10	0.13	−0.16	0.36	−0.06	0.05	−0.16	0.04	0.09	0.13	−0.17	0.35
Education	−0.69 ***	0.15	−0.98	−0.40	0.52 ***	0.06	0.41	0.64	−0.65 ***	0.15	−0.94	−0.35
Work	0.40 **	0.15	0.11	0.70	−0.25 ***	0.06	−0.36	−0.13	0.38 *	0.15	0.08	0.67
Pension	−0.13 **	0.04	−0.21	−0.04	0.10 ***	0.02	0.07	0.13	−0.12 **	0.04	−0.20	−0.03
ADL limitation	1.23 ***	0.16	0.91	1.56	−0.03	0.06	−0.15	0.09	1.23 ***	0.16	0.91	1.56
SRH	1.14 ***	0.21	0.72	1.56	−0.29 ***	0.08	−0.45	−0.13	1.12 ***	0.21	0.70	1.54
Chronic disease	0.71 ***	0.15	0.41	1.00	0.05	0.06	−0.06	0.17	0.71 ***	0.15	0.42	1.00
Depressive symptoms (2015)	0.46 ***	0.01	0.43	0.48	−0.03 ***	0.00	−0.03	−0.02	0.45 ***	0.01	0.43	0.47
Financial support	−0.10 *	0.05	−0.20	−0.01	0.19 ***	0.02	0.08	0.16	−0.09	0.05	−0.19	0.01
Social participation									−0.09 **	0.03	−0.15	−0.03
Constant	5.19 ***	0.91	3.39	6.98	4.07 ***	0.35	3.38	4.75	5.55 ***	0.92	3.74	7.36
R^2^					0.30				0.30			

* *p* < 0.05, ** *p* < 0.01, *** *p* < 0.001.

## Data Availability

The data that support the findings of this study are available at: https://charls.charlsdata.com/pages/Data/2018-charls-wave4/zh-cn.htmlh (accessed on 10 June 2022).
